# The Mortality of Child-Bed

**Published:** 1875-09

**Authors:** 


### THE MORTALITY OF CHILD-BED.

The following admirable abstract we take from the *British Medical Journal:*

We call attention to the means employed by Dr. Goodell, the professor of the diseases of women at the University of Pennsylvania, at the Preston Retreat for the treatment and prevention of puerperal diseases. In many respects, they are novel and revolutionary; they are, consequently, the better fitted for opening up of the system at present adopted for the management of parturient woman. Time and wider experience will prove whether they are founded on correct principles. The author did not intend to publish until he had completed his one thousand cases. As yet, he has only seven hundred and fifty-six. The mortality was only six; three from puerperal causes. The following extracts from his pamphlet seem to be of sufficient interest to justify reproduction.

The institution contains twenty beds, divided amongst four wards, five in each ward. The cubic capacity is about 1,800 feet for each bed. About one hundred married women are delivered yearly. They are admitted, on an average about sixteen days previously to confinement, and allowed to stay a month; they, however, generally only remained about eighteen days. The air admitted into the rooms is heated in the basement, and ventilation is maintained by a small jet of gas in the old-fashioned fireplace. Outside the hospital, puerperal fever was rife of late years. In Philadelphia and the city of New York, the mortality from puerperal causes (acknowledged as such) has been as high as one in forty-five, amongst all classes alike; if anything, more amongst the wealthier. The wards are used in rotation, one always being kept vacant for about two or three weeks. When a ward is emptied, the doors and windows are kept constantly opened until it is again used; and the whole of the walls, beds, furniture, and floors, are scrubbed down with carbolized soap, and then mopped over with a wet solution of carbolic acid. No water is allowed to be used to the floors until the ward is emptied again. The nurses belonging to the ward go off duty for a week when it is closed, and go through a thorough system of purification. The beds are of straw, which are changed with each patient; the blankets and bedclothes being boiled in water with a small quantity of carbolic acid added. The feathers of the pillows and bolsters are only baked once a year, unless they should become soiled, or have been used by the patient whose convalescence has been retarded. Every woman has a bath at least once a week before delivery. Any indication of enfeebled health is at once treated with quinine, steel, and phosphoric acid. Headaches and sleeplessness are dealt with by warm baths and large doses of bromide of potassium. The bowels are kept relaxed and purged. As soon as labor begins, the patient is put in a warm bath. The membranes are generally ruptured artificially. The second stage is never allowed to be prolonged, the forceps or vectis being used. The placenta is removed by Credè's method as soon as possible after delivery. The umbilical cord is not tied before it is cut. The blood and gelatin of the cord are "stripped" out as much as can be; and, when bleeding has ceased, it is tied. No binder is placed round the child; nor is the cord touched, but left to lie flaccid and loose on the abdominal walls. It dries up without any smell, and peels off without leaving any raw stump. Out of five hundred infants, not one has had a sore navel or an umbilical hernia.

Ergot is not given as an oxytoxic; but as soon as the head comes to press on the perinæum, a drachm is given. Should the perinæum be torn, it is sewn up at once with silver sutures. A cylindrical compress is applied just about the fundus, and a tight binder applied for twelve hours, when it is removed, and not used again. The patients are confined on a delivery-bed, and wheeled into the ward, and removed to their beds. In not a single instance has flooding ever been caused by this muscular movement; if anything, it has rather tended to excite uterine contraction than otherwise. The next day after delivery, the woman slips out and sits in a chair whilst her bed is made; this is repeated once or twice a day until the fourth or fifth day, at which time she may get up, dress herself, and do what she likes. No patient is forced to leave her bed; but the force of example is so great, that most do. After-pains are immediately removed by quarter-grain doses of morphia, given every hour until relief is obtained. If they be very obstinate, ten grains of quinine are given every six hours until the ears ring. It is an invaluable remedy in these cases. There are no bed-pans; vaginal injections are employed. Every woman washes herself daily with carbolized soap and a pad of fine oakum. No nurse, except for some special reason, is ever allowed to wash the woman's person. If the lochia be offensive, she is taken out of bed more frequently, and placed on the chair. Should this not succeed, a vaginal injection is then used.

Whenever the lochia are offensive, or the pulse over ninety, or the temperature higher than natural, or there are pelvic pains—in fact, when any untoward symptom appears—quinine is given, from six to ten grains every four hours until the ears ring. In addition, for abdominal pains, large doses of morphia are given, and the whole abdomen is painted with iodine, and a poultice applied over it. The canonical purge is never given. As soon as the patient feels srtong enough after getting up, she takes a warm bath.

The reason assigned for not using the bed-pan is, that the recumbent position tends to retain the utero-vaginal tract the putrescent discharges which are recognized by all authors as a great cause of the autogenetic variety of puerperal diseases. Besides, through the swollen condition of the parts, a putrid clot may be retained in any part of the passages ; even injection is not able ordinarily to dislodge it. The exertion necessary, and the position in which the woman places herself, in order to use the ordinary chamber-vessel, are a very effectual remedy to rid the uterus or vagina of any clots and putrescent discharges. Again, slipping into the chair two or three times a day is not only an excellent deodorant, but it enables the bed to be thoroughly aired. This, Dr. Goodell believes, is especially necessary for a lying-in hospital.

The writer hazards the assertion that there is a form of puerperal septicæmia not necessarily accompanied by putrid lochia—at least, not appreciably so—but indicated by a high temperature, rapid pulse, complete anorexia, heavy sweats, and later, by herpes labialis, which steadily resists treatment until the patient is made to get up. This we have seen several times ourselves, and we can bear witness to the truthfulness of the description, and have found that the only treatment consisted in taking the patient out of bed, when, by the second or third day, the whole of the symptoms disappeared. Examination of the vagina, abdomen, and chest, revealed nothing to account for it. When a recumbent position was strictly enforced during the first five days, we found that not only did the discharges become generally offensive, but in every case there was a rise of temperature, amounting to about one degree, although the pulse was not affected. We came to the conclusion that the retention of the fœtid discharges was the cause, and adopted the system of bed-chamber with the best results. The dorsal decubitus, Dr. Goodell, as also do many of the American writers, thinks, tends to a passive congestion of the uterus, and to engorgement of the greatly hypertrophied placenta in particular. The tight binder, continued for some days, is also thought to add to it still more by pressing on the abdominal vessels and retarding the circulation. Milk-fever he ignores, except in the rarest of instances. Purges, he considers, disturb the equilibrium, promote the absorption of septic matter, and act as hemorrhages do in labor, by increasing the activity of the absorbents. The appearance of septic poisoning on the third or fourth day is no mere coincidence; it is really cause and effect. Two cases the author states that he has seen to be directly due on a purge. Quinine should be always pushed to cinchonism whenever there are any symptoms of septic poisoning. Its power in producing absorption of the uterus, and preventing coagula from becoming detached, is esteemed to be very high.

Dr. Goodell believes that one of the reasons why the statistics of lying-in hospitals can never compete with private practice is, that the former are reliable, the latter not. His experience closely corresponds with Dr. Matthews Duncan's, and with that of all who have taken the trouble to investigate the matter thoroughly. It cannot be too strongly urged that the mortality of child-bed is much higher than what is generally stated, and that the ailments arising from it are of a more serious nature, and more frequent, than most medical practitioners suppose. It is to be hoped that the lying-in charities of London will not always remain the monopoly of nurses and midvives, but will be available for the training of medical students, whose present system of practical instruction is of the poorest description.—*Missouri Clinical Record.*

